# Gatekeepers and Gatecrashers of the Symplasm: Cross-Kingdom Effector Manipulation of Plasmodesmata in Plants

**DOI:** 10.3390/plants14213285

**Published:** 2025-10-27

**Authors:** Zhihua Li, Yonghong Wu, Xiaokun Liu, Muhammad Adnan

**Affiliations:** 1 College of Bioscience and Biotechnology, Hunan Agricultural University, Changsha 410128, China; lizhihua@stu.hunau.edu.cn (Z.L.); wuyonghong@stu.hunau.edu.cn (Y.W.); 2 Jiangxi Provincial Key Lab of Plant Germplasm Resources Innovation and Genetic Improvement, Lushan Botanical Garden, Chinese Academy of Sciences, Jiujiang 332900, China

**Keywords:** plasmodesmata, pathogen effectors, effector mediated PD modulation, symplastic transport, immune suppression, plant-microbe interaction

## Abstract

Plasmodesmata (PD) are dynamic nanochannels interconnecting plant cells and coordinating development, nutrient distribution, and systemic defense. Their permeability is tightly regulated by callose turnover, PD-localized proteins, lipid microdomains, and endoplasmic reticulum (ER)–plasma membrane (PM) tethers, which together form regulatory nodes that gate symplastic exchange. Increasing evidence demonstrates that effectors from diverse kingdoms—fungi, oomycetes, bacteria, viruses, viroids, phytoplasmas, nematodes, insects, parasitic plants, and symbiotic microbes—converge on these same nodes to modulate PD gating. Pathogens typically suppress callose deposition or destabilize PD regulators to keep channels open, whereas mutualists fine-tune PD conductivity to balance resource exchange with host immunity. This review synthesizes current knowledge of effector strategies that remodel PD architecture or exploit PD for intercellular movement, highlighting novel cross-kingdom commonalities–callose manipulation, reprogramming of PD proteins, lipid rewiring, and co-option of ER-PM tethers. We outline unresolved questions on effector–PD target specificity and dynamics, and identify prospects in imaging, proteomics, and synthetic control of PD. Understanding how effectors reprogram PD connectivity can enable engineering of crops that block pathogenic trafficking while safeguarding beneficial symbioses.

## 1. Introduction: Plasmodesmata as Dynamic Communication Hubs

### 1.1. PD Structural and Regulatory Overview

Plants maintain a living continuum of cytoplasm via plasmodesmata (PD)—membrane-lined, ER-tethered nanochannels that coordinate development, resource allocation, and systemic signaling by permitting selective symplastic exchange between cells [1]. PD permeability (size-exclusion limit, SEL) is tuned within seconds to hours by callose turnover at the PD neck through the antagonistic action of callose synthases (CalS/GSL) and β-1,3-glucanases. PD permeability is further modified by dedicated PD regulators including PD-located proteins (PDLPs), lipid-raft remorins, and ER–plasma membrane (PM) tethers such as SYTA (Synaptotagmin A) [2,3]. Mounting evidence shows that pathogens and mutualists exploit these regulatory nodes—directly at PD or indirectly through upstream signaling—to recalibrate SEL and thereby maintain, widen, or strategically restrict symplastic connectivity across host tissues.

For instance, the oomycete effector RxLR3 from *Phytophthora brassicae* binds PD-localized callose synthases CalS1/2/3, reduces PD callose, and enhances cell-to-cell trafficking in *Arabidopsis* [4], while the poplar rust *Melampsora larici-populina* protein Mlp37347 accumulates at PD, lowers callose, and increases PD flux with measurable gains in host susceptibility [5]. Conversely, plants integrate salicylic-acid and MAMP (microbe associated molecular pattern) signals through PDLPs (e.g., PDLP5/6) to stimulate callose and close PD; perturbing PDLP complexes or their interactor NHL3 shifts the system toward an open-PD state permissive for spread [6,7].

A second, widely used tactic is to hijack host trafficking to PD and remodel their architecture. Viral movement proteins (MPs) from diverse families localize to PD, bind viral genomes, remodel actin/ER scaffolds, and recruit ER–PM contact sites via SYTA; these activities transiently raise SEL or even build tubules that ferry RNPs (ribonucleoproteins)/virions between cells [8,9,10,11]. In parallel, several bacterial type III effectors are able to move from initially infected cells into neighbors via PD, while others destabilize PD regulators (e.g., PDLPs), collectively undermining PD-based immunity and facilitating intercellular colonization [12,13].

### 1.2. Biological and Physiological Roles of PD Regulation

Beyond pathogen entry, PD regulation underlies core biological and physiological processes: PD coordinate development and resource allocation but are rapidly reprogrammed during stress to balance communications and containment. Non-microbial partners reshape symplastic routes: phloem-feeding insects (aphids, whiteflies, leaf-/planthoppers) deliver salivary effectors that dampen elicitor signaling and interfere with rapid sieve-tube sealing. By modulating callose accumulation at sieve plates and pore-plasmodesma units (PPUs) between companion cells and sieve elements, they indirectly influence symplastic connectivity and the vascular movement of associated microbes [14,15]. Phytoplasmas—obligate phloem dwellers vectored by these insects—encode effectors (e.g., SAP11, SAP05) that rewire host development and defense; although direct PD targets are still emerging, phytoplasma infections correlate with altered callose dynamics in sieve pores that facilitate systemic spread [16]. Viroids, protein-free RNA pathogens, likewise move through PD using host factors, illustrating how PD regulation alone can determine invasion success [1].

Multicellular parasites further demonstrate PD plasticity: plant-parasitic nematodes induce giant cells/syncytia that become hyperconnected to surrounding tissues; this entails profound remodeling of PD density and callose homeostasis to sustain phloem unloading toward the feeding site [17,18]. Parasitic plants such as *Cuscuta* (dodder) form interfaces with hosts featuring PD-like cell–cell connections through which proteins, mRNAs, and even pathogens traffic bidirectionally, effectively co-opting symplastic channels at the haustorial bridge [19,20].

On the beneficial side, symbiotic microbes—including arbuscular mycorrhizal (AM) fungi and rhizobia—must integrate with host signaling without triggering immune PD closure; colonization correlates with regulated callose turnover and systemic cues that likely reset PD gating to balance nutrient exchange with surveillance [21,22].

### 1.3. Outline and Roadmap of the Review

**Figure 1:**
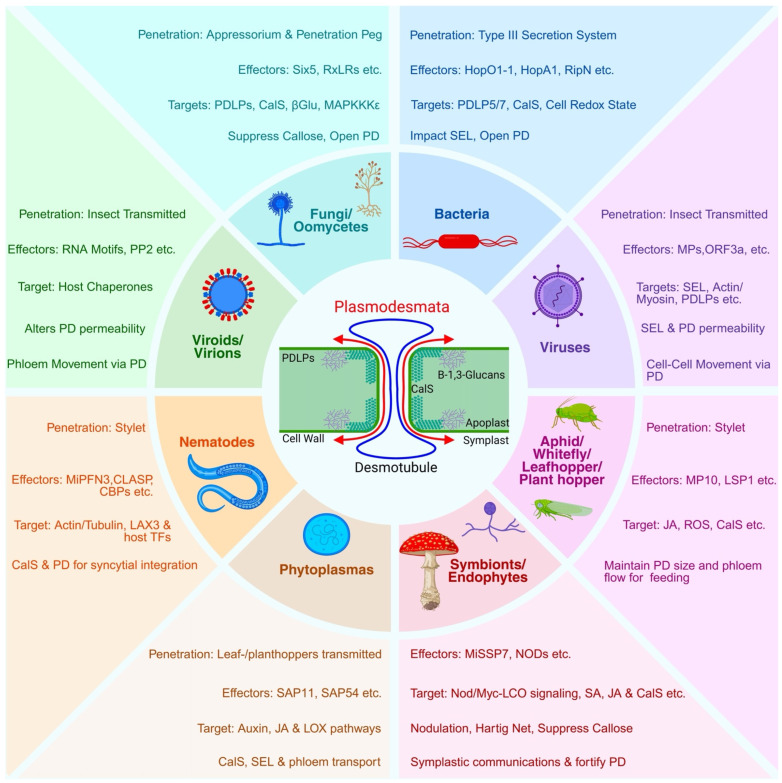
Effector strategies used by insects, microbes and mutualists to alter plasmodesmata: a cross-kingdom view of symplastic connectivity. This schematic summarized how plant-associated organisms—including insects, microbial pathogens and symbionts—manipulate PD to enable colonization, mobility or mutualistic exchange. The central panel shows PD architecture with key regulators such as PDLPs and CalS, which control permeability via callose deposition. The desmotubule is an ER-derived tubule that traverses each PD and links the ER of adjacent cells. ER-PM tethers are protein complexes that connect the ER to the PM at contact sites, coordinating lipid/Ca^2+^ exchange and often concentrating at PD. Surrounding panels depict major biotic agents—fungi, bacteria, viruses, nematodes, insects, phytoplasmas, viroids/virions, and symbionts/endophytes—indicating their penetration mode (e.g., appressorium, stylet, secretion systems), Key effectors (e.g., RxLRs, Hop effectors, movement proteins), PD relevant targets (CalS, PDLPs, actin, redox regulators) and outcomes such as suppressed callose, rewired SA/JA/auxin signaling, and enhanced symplastic transport. Briefly, the panels highlight cross-kingdom convergence: effectors either act directly on PD components or reprogram host signaling to keep PD open, dampen immunity and promote systemic spread or beneficial communication. Short description of individual panels (clockwise): **Fungi/Oomycetes:** Enter via an appressorium/penetration peg and deliver effectors such as RxLRs/Six5 that target PDLPs, CalS, β-1,3-glucanases, resulting in callose suppression and PD opening. **Bacteria:** Inject Type-III secreted effectors (e.g., HopO1-1, HopA1, RipN) that modulate PDLPs and CalS, increase the SEL, and maintain PD openness. **Viruses:** Often transmitted; movement proteins and auxiliary ORFs remodel actin/myosin and PLDPs to raise SEL and enable cell-to-cell spread. **Aphid/Whitefly/Leafhopper/Planthopper:** Deliver salivary effectors (e.g., MP10, LSP1) via the stylet, influencing JA/ROS signaling and CalS to keep PD/PPU corridors open for feeding and vectoring. **Symbionts/Endophytes:** Release MiSSP7 and Nod/Myc-LCO signals to tune SA/JA pathways and CalS, suppress callose, and strengthen symplastic communication (e.g., nodulation/Hartig net). **Phytoplasmas:** Insect-borne; SAP11/SAP54 effectors reprogram auxin, JA, CalS and LOX to alter SEL and phloem transport for systemic movement. **Nematodes:** Secrete MiPFN3/CLASP/Ca^2+^-binding proteins through stylet that target host actin/tubulin, LAX3, and transcription, reprogramming CalS and PD for syncytial integration. **Viroids/Virions:** Insect-assisted transmission; RNA motifs recruit PP2-like host chaperones to adjust PD permeability and enable phloem translocation.

This review synthesizes how effectors from fungi, oomycetes, bacteria, viruses, viroids/virions, phytoplasmas, phloem-feeding insects, nematodes, symbiotic microbes, and parasitic plants converge on PD control points—CalS/β-1,3-glucanase enzymes, PDLP-centered immune hubs, membrane microdomains (remorins), and ER–PM tethers (SYTA)—to reprogram plant cell–cell connectivity (see Table 1 for cross-kingdom effector mechanisms). Section 2 covers microbial strategies—fungal/oomycete, bacterial, viral, and viroid effectors—that manipulate callose turnover and PD architecture; it also considers non-microbial partners—including phloem-feeding insects, phytoplasmas, nematodes, and parasitic plants—that reshape symplastic routes. Section 3 examines beneficial symbioses (AM fungi and rhizobia) and how hosts modulate PD to accommodate partners while maintaining surveillance (as illustrated in Figure 1). Section 4 integrates cross-cutting mechanisms, highlights outstanding questions, and outlines opportunities to engineer PD-centered resilience.

## 2. Pathogen Effector Strategies at Plasmodesmata

### 2.1. Fungi

Fungal pathogens have repeatedly evolved effectors that manipulate PD to secure nutrient access and precondition neighboring cells for colonization. A well-studied case is the poplar rust effector Mlp37347, which accumulates at PD, lowers callose, and increases molecular flux, actively opening the PD gate [5]. In *Fusarium oxysporum*, the paired effectors Avr2 and Six5 interact at PD to expand the SEL and promote cell-to-cell spread of Avr2 and other effectors. Strikingly, this occurs without altering callose levels, suggesting a callose-independent gating mechanism [23,24].

Hemibiotrophs illustrate another strategy: pre-invasion priming. In *Magnaporthe oryzae*, effectors such as PWL2 and BAS1 are secreted into the biotrophic interfacial complex (BIC) and move through PD into uninvaded cells ahead of hyphal entry, staging compatibility in advance [25,26]. Disruption of BIC organization (e.g., RBF1 mutants) reduces effector mobility, linking secretion hubs with symplastic spread [26].

Other fungi reveal convergent tactics. In *Colletotrichum higginsianum*, hypermobile proteins (ChEC127, ChEC132) enhance PD flux of co-expressed reporters [27]. In *Fusarium graminearum*, fusaoctaxin A promotes intercellular spread while downregulating callose synthases and PD-associated genes, favoring PD openness [30,31]. The lipopeptide gramillin similarly suppresses callose during infection [32].

Biotrophic smuts provide mechanistic contrast. In *Ustilago maydis*, the enzyme effector Cmu1 spreads intercellularly—likely via PD—to divert chorismate from salicylic acid (SA) biosynthesis, suppressing immunity and indirectly sustaining PD permeability [28].

Together, these studies reveal two major fungal strategies: direct PD gating (e.g., Mlp37347, Avr2/Six5, ChEC127/132) and pre-invasion priming via PD transit (e.g., PWL2/BAS1, Cmu1), underscoring PD as a central vulnerability in fungal pathogenesis.

### 2.2. Oomycetes

Oomycetes such as *Phytophthora* spp. and downy mildews deploy RxLR effectors that target PD either by directly manipulating gating or by moving intercellularly to precondition host tissues. A clear example is *P. brassicae* RxLR3, which binds PD-localized callose synthases (CalS1/2/3), reduces callose, and thereby enhances symplastic trafficking in Arabidopsis [4]. This establishes callose suppression at PD as a key virulence node. Broader surveys suggest RxLR proteins frequently converge on callose metabolism or PD residents to weaken PD-based immunity [40].

In downy mildews, PD also serve as conduits for effector mobility. The *Hyaloperonospora arabidopsidis* effector HaRxL77 moves between cells in planta and promotes infection, consistent with earlier catalogs of mobile ATR/RxLR proteins [36,37]. Although direct PD targets remain unresolved, these findings support a model in which effectors spread ahead of hyphae to dampen defenses and adjust local physiology before haustorium formation.

Other effectors reshape trafficking pathways that intersect with PD control. The atypical RxLR PsAvh181 from *Phytophthora sojae* localizes to the PM and inhibits SNARE/NSF machinery, suppressing secretion of apoplastic defense proteins and indirectly preventing defense-induced PD closure [38]. Similarly, *Phytophthora infestans* RxLR repertoires highlight vesicle trafficking as a major host process under effector control, potentially influencing delivery of CalS and β-1,3-glucanases to PD [4].

In sum, oomycete effectors act through two complementary strategies: direct PD gating via callose synthase interference (e.g., RxLR3) and PD-assisted intercellular mobility (e.g., HaRxL77), supplemented by indirect modulation of host secretion and vesicle pathways that influence PD permeability.

### 2.3. Bacteria

Bacterial pathogens also exploit PD to promote infection. *Pseudomonas syringae* type III effectors (T3Es) both target PD regulators and move intercellularly to extend their reach. The best-characterized example is HopO1-1, which interacts with and destabilizes PD-located proteins (PDLPs). By eroding PDLP-mediated callose deposition, HopO1-1 undermines the PD-closing immune module and drives tissues toward an “open PD” state permissive for bacterial spread [12].

Effector mobility through PD provides a complementary strategy. In *Nicotiana benthamiana*, at least a dozen *P. syringae* DC3000 effectors—including HopAF1 and HopA1—were shown to cross into neighboring cells, with mobility inversely proportional to protein size. Importantly, overexpression of PDLP5 or PDLP7, or elicitation with the MAMP flg22, restricted this spread by triggering callose-dependent PD closure, linking pattern-triggered immunity directly to the containment of bacterial effector movement [13]. These findings establish PD as legitimate routes for effector dissemination and highlight PDLPs as central plant countermeasures.

Beyond direct PD targeting and mobility, T3Es also manipulate upstream processes that intersect with PD regulation. Many suppress defense signaling cascades, cytoskeletal dynamics, or membrane trafficking pathways that govern delivery of callose synthases and β-1,3-glucanases to PD [40,71]. Enhanced PD closure via PDLPs is increasingly recognized as an antibacterial defense mechanism; for instance, PDLP7, like PDLP5, promotes callose accumulation and restricts intercellular trafficking when overexpressed [72].

Thus, bacterial strategies at PD are two-pronged: neutralizing PD immune gatekeepers (e.g., HopO1-1 targeting PDLPs) and exploiting PD as conduits for effector spread (HopAF1, HopA1), countered by host surveillance that enforces PD closure.

### 2.4. Virus

Plant viruses depend on movement proteins (MPs) to exploit PD and spread between cells. Two strategies predominate: non-tubule movement, in which MPs enlarge the SEL and traffic viral ribonucleoprotein (RNP) complexes; and tubule-guided movement, in which MPs assemble PD-spanning tubules that conduct virions directly. In both modes, MPs recruit host endomembrane and cytoskeletal machineries, engage ER–PM contact sites, and counter callose-mediated closure [10,11].

The archetype is *Tobacco Mosaic Virus* (TMV) MP, a 30kD superfamily member that binds viral RNA, traffics along ER/actin, and increases SEL by remodeling PD. TMV MP interacts with SYTA, an ER–PM tether required for movement of diverse viruses, highlighting a conserved host dependency [9,11,73]. Potexviruses such as *Potato Virus X* (PVX) encode a triple gene block (TGB). TGBp1 binds RNA and elevates SEL, while TGBp2/3 remodel ER membranes to deliver RNPs to PD. Host remorins (e.g., REM1.3) antagonize this remodeling, illustrating a molecular tug-of-war [71,74]. Potyviruses employ P3N-PIPO to anchor the movement complex at PD, recruiting CI helicase and replication vesicles via P3/6K2. P3N-PIPO interacts with host PCaP1 to remodel actin; disruption of either protein blocks intercellular spread [10,11].

For tubule-guided movement, MPs from nepo-, como-, and caulimoviruses (e.g., GFLV 2B, CaMV P1) polymerize into tubules that replace PD membranes, allowing virion passage independent of SEL [11].

Thus, viral MPs function as specialized PD remodelers: widening channels for RNPs, building tubules for virions, and co-opting host trafficking while evading callose-based defenses. These strategies explain how diverse viruses converge on PD as critical gateways for systemic infection.

### 2.5. Viroids and Virions

Viroids are non-encapsulated, circular RNAs that move cell-to-cell and systemically through PD without encoding proteins. Their intercellular trafficking is guided by conserved RNA tertiary motifs that serve as “addresses.” In pospiviroids, the C-loop and loop-E elements direct subcellular targeting and symplastic passage independently of translation [43,75,76]. For *Potato Spindle Tuber Viroid* (PSTVd), the host RNA-binding protein Virp1 recognizes the C-loop and partners with importin-α4 to mediate nuclear import; a prerequisite for replication that also positions viroid RNPs for PD-mediated export [43,44]. Crossing tissue boundaries (epidermis–mesophyll–bundle sheath) requires boundary-specific motifs and host factors; underscoring that RNA structure alone programs PD transit [44]. Systemic spread occurs via PD at PPUs linking companion cells and sieve elements, with viroid infection often altering callose homeostasis to sustain movement in the phloem stream [77,78].

Viroids also influence PD gating indirectly through immunity. Defense responses elevate callose and restrict RNA movement, whereas successful viroids favor conditions that reduce callose or increase β-1,3-glucanase activity, maintaining a permissive SEL. Transcriptomic and genetic data link RNA silencing and hormone pathways to this balance [44,79]. Replication itself relies on host polymerases and ribozymes, but the critical virulence step is RNA–host factor interplay at PD [80,81].

Virions, in contrast, traverse PD as intact particles only when tubule-forming MPs assemble conduits through the wall. In caulimoviruses and nepo/comoviruses, MPs polymerize into PD-spanning tubules that ferry virions across cells [45,46]. Unlike the non-tubule RNP pathway, virion spread depends on these pre-built PD passages, highlighting that the particle itself plays no active role in gating.

### 2.6. Phytoplasmas

Phytoplasmas are phloem-restricted Mollicutes that secrete small effectors from sieve elements, many of which are small enough to unload into companion cells via PPUs and spread through sink tissues [16,82,83]. Although direct PD targets are only beginning to emerge, converging evidence indicates that phytoplasmas indirectly regulate PD gating by reprogramming hormone signaling, altering phloem homeostasis, and modulating callose dynamics.

Among characterized effectors, SAP11 destabilizes class II TCP transcription factors, altering jasmonate/auxin signaling and meristem identity in ways associated with permissive PD states [47]. SAP05 promotes degradation of SPL/GATA transcription factors, maintaining juvenile, sink-like tissues that favor unloading [16]. SAP54/PHYL1 induces phyllody by degrading MADS-box floral regulators, enhancing vector attraction and systemic colonization [84,85]. The small peptide TENGU (~4.5 kDa) perturbs auxin signaling and moves into meristems, consistent with PD-mediated spread [48,86].

Phytoplasma infections are tightly linked to callose remodeling. Excessive callose deposition at sieve plates reduces pore diameter and sucrose translocation in potato, altering development [49]. In pear and peach, callose accumulates differently than in apple, underscoring host-specific PD regulation [87]. Loss of the sieve element-specific callose synthase CalS7 in Arabidopsis increases susceptibility to Chrysanthemum Yellows phytoplasma and perturbs sugar transport [88]. Infection also triggers Ca^2+^ influx and occlusion by callose/protein plugs, directly affecting PD/PPU permeability [89]. Ultrastructural studies reveal ER remodeling and altered expression of ER tethers near sieve plates, suggesting specialized PPUs as corridors for effector exchange [90].

Together, phytoplasma effectors maintain sink status (SAP11/05/54/TENGU), suppress defenses, and modulate callose gating at sieve plates and PD, ensuring sustained symplastic connectivity and systemic colonization.

### 2.7. Nematodes

Sedentary endoparasitic nematodes remodel roots into giant cells (*Meloidogyne*) or syncytia (*Heterodera/Globodera*), which become highly connected to surrounding tissues through dense PD and dynamic callose turnover. Limiting callose degradation restricts syncytium size, highlighting the importance of controlled callose removal [91]. In rice, sucrose delivery to *Meloidogyne graminicola* giant cells depends primarily on PD, with callose correlating with gall sink strength [17].

Nematode effectors target PD by manipulating callose metabolism or reprogramming host development. The cyst nematode effector 30C02 binds PR2, a β-1,3-endoglucanase, interfering with callose degradation and defense [92]. *Heterodera schachtii* effectors 19C07 and 10A06 rewire physiology: 19C07 targets the auxin influx carrier LAX3 to enhance wall remodeling and PD conductance [52], while 10A06 binds spermidine synthase to elevate polyamine flux and suppress SA–linked PD closure [93].

Root-knot nematodes use additional effectors. The Ca^2+^-binding protein Mi-CRT suppresses PAMP-triggered callose and ROS in Arabidopsis, creating a permissive symplastic environment [50]. Other effectors modulate hormone and ROS signaling (e.g., MiISE/MiMIFs), reinforcing sink identity and countering PD closure [51].

Molecular mimicry further supports connectivity. CLE-like peptides mimic plant CLE signals to maintain meristematic states in feeding sites, sustaining PD density [94]. Chorismate mutases divert chorismate from SA biosynthesis, reducing SA-driven PD closure [95].

Thus, nematodes employ two complementary PD strategies: callose-centric control (30C02, Mi-CRT) and developmental/metabolic reprogramming (19C07, 10A06, CLEs), together ensuring high symplastic flux into feeding sites.

### 2.8. Insects

Phloem-feeding *Hemiptera* manipulate PD and sieve tube gating to sustain sap ingestion and assist microbe transmission. Rapid occlusion of sieve pores and PPUs depends on Ca^2+^ influx and callose deposition [1,96]. Aphids counter this with watery saliva rich in Ca^2+^-binding proteins that prevent occlusion and maintain symplastic conductivity [97,98].

Characterized aphid effectors further suppress immunity. *Myzus persicae* Mp10 dampens PTI (pattern triggered immunity)-associated ROS [53,54], Mp55 promotes performance and reduces defenses [54], while C002/Mp1/Mp2 families are essential for prolonged feeding [55,99]. Because SA and ROS promote callose-based PD closure, these effectors indirectly sustain open PD states.

Whiteflies secrete effectors such as Bsp9 and Bt56 that modulate WRKY-centered defense networks converging on callose gating [56,57,58], while BtE3 alters SA/JA cross-talk, influencing PD closure [59].

Planthoppers and leafhoppers also secrete Ca^2+^-binding effectors. In the brown planthopper *Nilaparvata lugens*, NlSEF1 binds Ca^2+^, suppressing ROS and callose deposition, while salivary calmodulin has a similar role [60,61]. Leafhoppers such as *Nephotettix cincticeps* deploy secreted Ca^2+^-binding proteins, consistent with a conserved anti-occlusion strategy [62].

Overall, insect saliva contains two effector classes: (i) Ca^2+^-binding proteins that block rapid occlusion, and (ii) immune modulators that suppress SA/JA/ROS pathways linked to PD closure. By sustaining low Ca^2+^ and limiting callose deposition, phloem feeders maintain open PD/PPU corridors for extended feeding and vectoring of associated microbes.

### 2.9. Parasitic Plants

Parasitic angiosperms establish interspecific plasmodesmata (iPD) at haustorial interfaces, forming graft-like connections for macromolecule and signal exchange [100,101]. In *Cuscuta* spp., iPD mediate bidirectional transfer of thousands of RNAs and proteins [19,102] and even transmit defense signals and pathogens between connected hosts [103]. This flux depends on cell-wall remodeling and sustained low callose states [63,64].

A defining effector layer involves microRNAs. In *C. campestris*, haustorium-induced miRNAs accumulate at the interface, target host transcripts in an AGO1-dependent manner, and promote parasitism [104]. Dedicated promoters underlie this specialized sRNA program [105]. Conversely, host-to-parasite RNA flow enables host-induced gene silencing (HIGS), confirming iPD as functional RNA conduits [106].

Mechanistically, iPD formation and maintenance couple localized wall softening with suppression of callose deposition, while hosts counter with wall fortification and callose barriers [63,100]. Functional openness is evident from herbivory-induced systemic signals transmitted through *Cuscuta* bridges [103]. Hormones and peptides also act as symplastic effectors: Orobanchaceae parasites deliver cytokinins to remodel host roots, consistent with iPD-mediated hormone flux [107].

Thus, parasitic plants (i) establish iPD through wall remodeling, (ii) deploy sRNA effectors to suppress host defense, and (iii) modulate callose gating to sustain symplastic connectivity. Outstanding questions include identifying PD resident targets of parasitic proteins and testing whether iPD employ PDLPs or unique tethers, sharpening parallels with microbe-induced PD modulation.

## 3. Symbiont Effector Strategies

Mutualistic microbes must establish compatibility while avoiding PD closure that would curtail nutrient exchange [108]. AM fungi exemplify this strategy through effectors that dampen pattern-triggered immunity and sustain low callose states [109]. The canonical AM effector SP7 enters host nuclei and interacts with the defense-related transcription factor ERF19 to suppress ethylene-linked defenses, thereby biasing tissues away from PD closure and maintaining symplastic conductance [69]. Mechanistically, SP7-mediated defense suppression has been demonstrated in *Medicago/Arabidopsis* systems and linked to improved colonization, providing a direct example of an AM effector that keeps PD conductive during accommodation [69]. LysM effectors such as RiSLM bind chitin oligomers to dampen chitin-triggered immunity and protect fungal hyphae from host chitinases; these actions reduce upstream ROS/SA signaling that otherwise promotes callose accumulation at PD neck regions. Notably, RiSLM is both highly expressed during symbiosis and necessary for colonization, and can polymerize in a chitin-dependent manner—properties that help explain durable suppression of callose-linked PD closure near colonization sites [110].

Additional AM effectors further stabilize a “low-callose, high-conductance” PD environment. The nuclear-localized effector RiNLE1 traffics to the host nucleus, binds histone H2B, and impairs H2B mono-ubiquitination to repress defense gene expression—events correlated with enhanced colonization and maintenance of symplastic exchange needed for arbuscule development [111]. Moreover, the crinkler effector RiCRN1 of *Rhizophagus irregularis* functions in arbuscule development, illustrating that AM fungi deploy multiple effector classes to coordinate developmental remodeling with immune attenuation, a combination that ultimately preserves PD permeability during nutrient transfer [112].

Ectomycorrhizal fungi and beneficial endophytes employ similar tactics. *Laccaria bicolor* MiSSP7 stabilizes JAZ repressors and blocks jasmonate signaling—a central antiherbivore/anti-microbe pathway—thereby indirectly maintaining PD openness and symplastic flow in colonized roots [67]. The root endophyte *Serendipita indica* secretes E5′NT, an ecto-5′-nucleotidase that hydrolyzes extracellular ATP to adenosine; by lowering apoplastic eATP—a danger signal that triggers Ca^2+^/ROS bursts and callose deposition. E5′NT suppresses defense outputs that would otherwise tighten PD, supporting sustained intercellular exchange during early symbiosis [70].

In rhizobia, Nod factor signaling provides a clear mechanistic link between symbiotic signaling and PD gating. Nod factor signaling transiently lowers callose at cortical PD to synchronize infection-thread progression with cell divisions in the inner cortex; restricting PD by hyperactivating callose synthases disrupts this coordination and nodule organogenesis. Primary and perspective studies show that callose-regulated symplastic communication coordinates root-nodule organogenesis with epidermal infection sites, with localized callose turnover at PD enabling spatiotemporal coupling across tissues [65]. Downstream of Nod-factor perception (NFR/NFP pathway), Ca^2+^ spiking and CCaMK/CYCLOPS signaling reprogram transcription and cytoskeletal dynamics; in parallel, PD-associated β-1,3-glucanases (e.g., MtBG2) fine-tune callose levels to define symplastic domains that guide infection threads and primordia development [113]. These studies provide direct evidence that symbiotic signals actively control PD callose turnover to coordinate developmental fields with microbial ingress.

Linking back to PD biology, these symbiont strategies converge on the same molecular switches that regulate PD aperture in defense and development: rapid callose synthesis/degradation at the PD neck, mediated by CalS/GSL enzymes and β-1,3-glucanases, and modulated by upstream immune and hormonal crosstalk. Reviews of PD callose homeostasis and wall microdomains emphasize that adjusting callose is the dominant route for tuning the PD SEL and symplastic flux—providing the mechanistic substrate that mutualists exploit to keep PD open without broadly disabling surveillance [114].

Thus, symbiotic microbes converge on a PD strategy distinct from pathogens: effectors attenuate immune and hormone pathways upstream of PD, biasing tissues toward high conductance, low-callose states that permit nutrient exchange and developmental reprogramming. Unresolved questions include whether symbionts directly target PD residents such as PDLPs or callose synthases (beyond indirect upstream signaling), and how β-1,3-glucanases are recruited spatiotemporally during accommodation—knowledge that is now within reach given expanding effector catalogs, PD proteomics, and cell-type-resolved symbiosis datasets.

## 4. Knowledge Gaps: Unresolved Mechanisms and Challenges

Despite recent progress, how effectors act at PD in vivo remains incompletely resolved. For many, direct PD targets—CalS, β-1,3-glucanases, PDLPs, ER–PM tethers, remorins, or receptor-like kinases—are still unknown, and conservation of these interactions across species is poorly defined. In numerous pathosystems, PD manipulation is inferred indirectly from immune or hormone reprogramming, making it difficult to distinguish primary PD targeting from downstream effects. To move beyond inference, the field needs cross-kingdom PD proteomics and effector-interactome mapping (fungi, bacteria, viruses, and mutualists) to reveal conserved host targets and evolutionary patterns of PD exploitation.

Quantitative assessment remains a bottleneck. Standardized assays for PD SEL and molecular flux that work across species and tissues are scarce, and spatial/temporal resolution is limited. PD at sieve element–companion cell interfaces behave differently from mesophyll or epidermal PD, yet rules for these specialized junctions are largely unknown. Practical advances include genetically encoded biosensors (Ca^2+^/pH/ROS), FRAP/FRET-based flux reporters, and simple reporter “ladders” to benchmark permeability across tissues and species; CRISPR/Cas functional analysis of PDLPs and other candidates—preferably multiplex and tissue specific—will clarify causal roles with minimizing overexpression artifacts.

At the structural level, we lack high-resolution views of PD remodeling under effector action. How desmotubules, lipid microdomains, and cell-wall matrices reorganize to permit or block traffic remains speculative. Future work should combine cryo-electron tomography with live-cell super-resolution (e.g., lattice light-sheet/STED) and correlative light–electron workflows to visualize PD remodeling in real time during effector activity.

Finally, PDs are difficult to isolate biochemically, and genetic redundancy complicates validation. Overcoming these hurdles—through proximity labeling at PD, inducible/optogenetic perturbations of callose enzymes, and harmonized quantitative pipelines—will be essential to establish causal links between specific effectors, PD components, and host phenotypes.

## 5. Future Prospects and Conclusions

Advancing PD biology requires linking molecules to mechanisms, structures, and phenotypes. A priority is target discovery: proximity labeling, crosslinking proteomics, and native PD fractionation with tagged effectors promise bona fide interactomes. In parallel, the field needs quantitative PD biophysics: shared reporter ladders for proteins and RNAs, defined RNA motifs, and rapid perturbations—such as optogenetic control of callose synthases and glucanases—to benchmark gating kinetics across species. High-resolution imaging, from cryo-ET to correlative light–electron microscopy, should reveal how desmotubules, wall porosity, and ER–PM contacts remodel during effector action. Single-cell and spatial omics can map PD programs in phloem versus mesophyll and track their dynamics across infection or symbiotic zones.

Translational opportunities are clear. Editing PDLPs, specific callose synthases/glucanases, remorins, and ER–PM tethers, combined with decoy peptides or nanobodies that sequester effector motifs, could reset PD set points: enforcing rapid closure against pathogens while permitting openness for nodules and arbuscules. Field-scale validation will be crucial, linking PD traits to microbiome assembly, vector behavior, carbon efficiency, and climate resilience.

Manipulating PD to increase openness could inadvertently facilitate long-distance movement of pathogens or their RNAs/proteins in transgenic plants. Conversely, enforcing tighter closure may impair development, carbon allocation, or beneficial symbioses. Off-target effects from editing PD regulators and pleiotropic impacts on wall mechanisms or Ca^2+^ signaling are plausible, as are ecological trade-offs such as altered vector transmission or microbiome balance. Mitigation should include reversible or tissue-specific control (chemically inducible/optogenetic switches), containment trials, and post-release monitoring focused on pathogen spread and yield quality trade-offs.

In conclusion, decoding and deliberately resetting PD gating offers dual dividends: durable disease resistance through smarter closure and optimized symbiosis through precise, reversible openness. These advances will elevate PD from a structural curiosity to an engineerable hub for plant health and productivity.

## Figures and Tables

**Table 1:** Cross-kingdom effector mechanisms targeting plasmodesmata for invasion, mobility, and symbiosis.

Organism Type	Example (Species)	Effector(s)	Target(s)/ Mechanism	Outcome/ Effect on PD	References
Fungi	*F. oxysporum*	Six5/Avr2	Alters SEL	Enhanced Intercellular movement	[23,24]
	*M. oryzae*	PWL2, BAS1	HIPP43 (heavy metal-binding isoprenylated protein)	Suppress local defense	[25,26]
	*M. larici-populina*	MLP37347	Upregulates β 1,3-glucanases, downregulates CalS	PD opening, cell-to-cell movement	[5]
	*C. higginsianum*	ChEC127, ChEC132	Indirect PD regulation	Systemic infection	[27]
	*U. maydis*	Cmu1	Indirect regulation for biotrophic interface	Cell-to-cell movement	[28,29]
	*F. graminearum*	Fusaoctaxin A	Suppress cell wall depositions	Suppress plant immunity	[30,31,32]
Oomycetes	*P. brassicae*	RxLR3	Binds CalS1-3	Suppresses callose, opens PD	[4]
	*P. viticola*	PvRXLR131	Interacts with BKI1	Suppress callose	[33]
	*P. infestans*	AVR1	Disrupts exocyst Sec5 & CalS	Suppresses callose, opens PD	[34]
		PexRD2	Interacts with MAPKKKε	Suppress immune signaling	[35]
	*H. arabidopsidis*	HaRxLs	Upregulates β 1,3-glucanases, downregulates CalS	Suppress callose, PD opening	[4,36,37,38]
	*P. parasitica*	PSE1	Modulates auxin signaling	Promote cell-to-cell flux	[39]
Bacteria	*P. syringae* pv. tomato DC3000	HopA1, HopAF1, HopO1-1	Degrades PDLP5/7	Increased symplastic flux via SEL changes and PD opening	[12,13,40]
	*R. solanacearum*	*RipN*	Alters cell redox state to suppress callose deposition	PD opening, bacterial movement	[41]
Viruses	TMV	MP30	Disrupts actin/ER network & PDLPs	Changes SEL to keep PD open	[9]
	CMV	MP	Actin cytoskeleton, formins	PD opening	[42]
	Potyviruses	P3N-PIPO	Suppress PD associated actin filaments & callose	SEL enlargement and PD opening	[10,11]
Viroid/Virions	PSTVd	Virp1	Interact with Loop motifs	Tissue-specific PD transit	[43,44]
	nepo/comoviruses	MPs	Polymerize into PD-spanning tubules	Bypass SEL limits	[45,46]
Phytoplasmas	Aster Yellows Witches’ Broom	SAP11	Destabilizes class II TCP, suppresses JA, Auxin signaling	Diminish callose at sieve pores and PD	[47]
	Onion Yellows Phytoplasma	TENGU	Suppress Auxin & JA pathways	Excessive callose deposition at sieve pores, reduced phloem transport	[48,49]
Nematodes	*M. incognita*	Mi-CRT	Calcium-binding effector, suppresses PAMP-triggered callose deposition	Suppresses callose to increase PD permeability	[50,51]
	*H. schachtii*	19C07, 10A06	Auxin transporter LAX3 and SPDS2	Callose degradation, PD reopening for syncytial integration	[52]
Aphids	*M. persicae*	MP10, Mp55	Modulates callose deposition	Keeps PD open for feeding facilitation	[53,54,55]
Whiteflies	*B. tabaci*	Bsp9, Bt56, BtE3	Targets WRKY33, alter s SA, JA	Modulates callose based gating, phloem flow to support feeding	[56,57,58,59]
Planthoppers/Leafhoppers	*N. lugens*, *N. cincticeps*	NlSEF1, CaM	Binds Ca^2+^, reduce H_2_O_2_ accumulation	Inhibit callose and ROS, keep PD/sieve pores open for feeding	[60,61,62]
Parasitic plants	*C. campestris*	miRNA	Cell wall remodeling	Modulation of PD gating and cell-wall architecture	[63,64]
Symbionts	Rhizobia	Nod effectors	Nod signaling	PD opening	[65,66]
	*L. bicolor* (ECM Fungi)	MiSSP7, MiSSP7.6	Stabilizes PtJAZ6, suppresses JA-and CalS	PD regulation, essential for colonization	[67,68]
	*R. irregularis* (AM Fungi)	SP7	Targets ERF19 TFs, suppresses immune signaling and callose	Enables symplastic nutrient transfer via PD	[69]
	*S. indica*	E5′NT	Enhances ATP induced Ca^2+^ influx, ROS, callose	Regulate callose homeostasis at PD	[70]

## Data Availability

No new data were created or analyzed in this study. Data sharing is not applicable to this article.

## References

[1] Chen J., Xu X., Liu W., Feng Z., Chen Q., Zhou Y., Sun M., Gan L., Zhou T., Xuan Y. (2024). Plasmodesmata Function and Callose Deposition in Plant Disease Defense. Plants.

[2] Bayer E.M., Benitez-Alfonso Y. (2024). Plasmodesmata: Channels under Pressure. Annu. Rev. Plant Biol..

[3] Tee E.E., Faulkner C. (2024). Plasmodesmata and Intercellular Molecular Traffic Control. New Phytol..

[4] Tomczynska I., Stumpe M., Doan T.G., Mauch F. (2020). A Phytophthora Effector Protein Promotes Symplastic Cell-to-cell Trafficking by Physical Interaction with Plasmodesmata-localised Callose Synthases. New Phytol..

[5] Rahman M.S., Madina M.H., Plourde M.B., Dos Santos K.C.G., Huang X., Zhang Y., Laliberté J.-F., Germain H. (2021). The Fungal Effector Mlp37347 Alters Plasmodesmata Fluxes and Enhances Susceptibility to Pathogen. Microorganisms.

[6] Li Z., Liu S.-L., Montes-Serey C., Walley J.W., Aung K. (2024). PLASMODESMATA-LOCATED PROTEIN 6 Regulates Plasmodesmal Function in Arabidopsis Vasculature. Plant Cell.

[7] Zanini A.A., Burch-Smith T.M. (2024). New Insights into Plasmodesmata: Complex ‘Protoplasmic Connecting Threads’. J. Exp. Bot..

[8] Levy A., Zheng J.Y., Lazarowitz S.G. (2015). Synaptotagmin SYTA Forms ER-Plasma Membrane Junctions That Are Recruited to Plasmodesmata for Plant Virus Movement. Curr. Biol..

[9] Yuan C., Lazarowitz S.G., Citovsky V. (2018). The Plasmodesmal Localization Signal of TMV MP Is Recognized by Plant Synaptotagmin SYTA. MBio.

[10] Morozov S.Y., Solovyev A.G. (2024). Mechanisms of Plant Virus Cell-to-Cell Transport: New Lessons from Complementation Studies. Front. Plant Sci..

[11] Alazem M., Nuzzi S.N., Burch-Smith T.M. (2025). Regulation of Cell-to-Cell Trafficking by Viral Movement Proteins. J. Exp. Bot..

[12] Aung K., Kim P., Li Z., Joe A., Kvitko B., Alfano J.R., He S.Y. (2020). Pathogenic Bacteria Target Plant Plasmodesmata to Colonize and Invade Surrounding Tissues. Plant Cell.

[13] Li Z., Variz H., Chen Y., Liu S.-L., Aung K. (2021). Plasmodesmata-Dependent Intercellular Movement of Bacterial Effectors. Front. Plant Sci..

[14] Walker G. (2022). Sieve Element Occlusion: Interactions with Phloem Sap-Feeding Insects. A Review. J. Plant Physiol..

[15] Li N., Lin Z., Yu P., Zeng Y., Du S., Huang L.-J. (2023). The Multifarious Role of Callose and Callose Synthase in Plant Development and Environment Interactions. Front. Plant Sci..

[16] Haider M.W., Sharma A., Majumdar A., Fayaz F., Bromand F., Rani U., Singh V.K., Saharan M.S., Tiwari R.K., Lal M.K. (2024). Unveiling the Phloem: A Battleground for Plant Pathogens. Phytopathol. Res..

[17] Xu L., Xiao L., Xiao Y., Peng D., Xiao X., Huang W., Gheysen G., Wang G. (2021). Plasmodesmata Play Pivotal Role in Sucrose Supply to Meloidogyne Graminicola-caused Giant Cells in Rice. Mol. Plant Pathol..

[18] Chen X., Li F., Wang D., Cai L. (2024). Insights into the Plant Response to Nematode Invasion and Modulation of Host Defense by Plant Parasitic Nematode. Front. Microbiol..

[19] Li T., Deng Y., Huang J., Liang J., Zheng Y., Xu Q., Fan S., Li W., Deng X., Zheng Z. (2022). Bidirectional mRNA Transfer between Cuscuta Australis and Its Hosts. Front. Plant Sci..

[20] Jhu M.-Y., Sinha N.R. (2022). Cuscuta Species: Model Organisms for Haustorium Development in Stem Holoparasitic Plants. Front. Plant Sci..

[21] Lepetit M., Brouquisse R. (2023). Control of the Rhizobium–Legume Symbiosis by the Plant Nitrogen Demand Is Tightly Integrated at the Whole Plant Level and Requires Inter-Organ Systemic Signaling. Front. Plant Sci..

[22] de Carvalho-Niebel F., Fournier J., Becker A., Arancibia M.M. (2024). Cellular Insights into Legume Root Infection by Rhizobia. Curr. Opin. Plant Biol..

[23] Blekemolen M.C., Cao L., Tintor N., de Groot T., Papp D., Faulkner C., Takken F.L. (2022). The Primary Function of Six5 of *Fusarium oxysporum* Is to Facilitate Avr2 Activity by Together Manipulating the Size Exclusion Limit of Plasmodesmata. Front. Plant Sci..

[24] Cao L., Blekemolen M.C., Tintor N., Cornelissen B.J., Takken F.L. (2018). The *Fusarium oxysporum* Avr2-Six5 Effector Pair Alters Plasmodesmatal Exclusion Selectivity to Facilitate Cell-to-Cell Movement of Avr2. Mol. Plant.

[25] Khang C.H., Berruyer R., Giraldo M.C., Kankanala P., Park S.-Y., Czymmek K., Kang S., Valent B. (2010). Translocation of Magnaporthe Oryzae Effectors into Rice Cells and Their Subsequent Cell-to-Cell Movement. Plant Cell.

[26] Nishimura T., Mochizuki S., Ishii-Minami N., Fujisawa Y., Kawahara Y., Yoshida Y., Okada K., Ando S., Matsumura H., Terauchi R. (2016). Magnaporthe Oryzae Glycine-Rich Secretion Protein, Rbf1 Critically Participates in Pathogenicity through the Focal Formation of the Biotrophic Interfacial Complex. PLoS Pathog..

[27] Ohtsu M., Jennings J., Johnston M., Breakspear A., Liu X., Stark K., Morris R.J., de Keijzer J., Faulkner C. (2024). Assaying Effector Cell-to-Cell Mobility in Plant Tissues Identifies Hypermobility and Indirect Manipulation of Plasmodesmata. Mol. Plant-Microbe Interact..

[28] Djamei A., Kahmann R. (2012). *Ustilago maydis*: Dissecting the Molecular Interface between Pathogen and Plant. PLoS Pathog..

[29] Tanaka S., Brefort T., Neidig N., Djamei A., Kahnt J., Vermerris W., Koenig S., Feussner K., Feussner I., Kahmann R. (2014). A Secreted *Ustilago maydis* Effector Promotes Virulence by Targeting Anthocyanin Biosynthesis in Maize. eLife.

[30] Jia L.-J., Tang H.-Y., Wang W.-Q., Yuan T.-L., Wei W.-Q., Pang B., Gong X.-M., Wang S.-F., Li Y.-J., Zhang D. (2019). A Linear Nonribosomal Octapeptide from Fusarium Graminearum Facilitates Cell-to-Cell Invasion of Wheat. Nat. Commun..

[31] Mapuranga J., Chang J., Zhang L., Zhang N., Yang W. (2022). Fungal Secondary Metabolites and Small RNAs Enhance Pathogenicity during Plant-Fungal Pathogen Interactions. J. Fungi.

[32] Brauer E.K., Bosnich W., Holy K., Thapa I., Krishnan S., Syed M., Bredow M., Sproule A., Power M., Johnston A. (2024). A Cyclic Lipopeptide from Fusarium Graminearum Targets Plant Membranes to Promote Virulence. Cell Rep..

[33] Lan X., Liu Y., Song S., Yin L., Xiang J., Qu J., Lu J. (2019). Plasmopara Viticola Effector PvRXLR131 Suppresses Plant Immunity by Targeting Plant Receptor-like Kinase Inhibitor BKI1. Mol. Plant Pathol..

[34] Du Y., Mpina M.H., Birch P.R., Bouwmeester K., Govers F. (2015). Phytophthora Infestans RXLR Effector AVR1 Interacts with Exocyst Component Sec5 to Manipulate Plant Immunity. Plant Physiol..

[35] King S.R., McLellan H., Boevink P.C., Armstrong M.R., Bukharova T., Sukarta O., Win J., Kamoun S., Birch P.R., Banfield M.J. (2014). Phytophthora Infestans RXLR Effector PexRD2 Interacts with Host MAPKKKε to Suppress Plant Immune Signaling. Plant Cell.

[36] Liu X., Bellandi A., Johnston M.G., Faulkner C. (2022). The *Hyaloperanospora arabidopsidis* Effector HaRxL77 Is Hypermobile between Cells and Manipulates Host Defence. bioRxiv.

[37] Fabro G., Steinbrenner J., Coates M., Ishaque N., Baxter L., Studholme D.J., Körner E., Allen R.L., Piquerez S.J., Rougon-Cardoso A. (2011). Multiple Candidate Effectors from the Oomycete Pathogen Hyaloperonospora Arabidopsidis Suppress Host Plant Immunity. PLoS Pathog..

[38] Wang H., Guo B., Yang B., Li H., Xu Y., Zhu J., Wang Y., Ye W., Duan K., Zheng X. (2021). An Atypical Phytophthora Sojae RxLR Effector Manipulates Host Vesicle Trafficking to Promote Infection. PLoS Pathog..

[39] Huang G., Liu Z., Gu B., Zhao H., Jia J., Fan G., Meng Y., Du Y., Shan W. (2019). An RXLR Effector Secreted by *Phytophthora parasitica* Is a Virulence Factor and Triggers Cell Death in Various Plants. Mol. Plant Pathol..

[40] Iswanto A.B.B., Vu M.H., Pike S., Lee J., Kang H., Son G.H., Kim J., Kim S.H. (2022). Pathogen Effectors: What Do They Do at Plasmodesmata?. Mol. Plant Pathol..

[41] Jeon H., Segonzac C. (2023). Manipulation of the Host Endomembrane System by Bacterial Effectors. Mol. Plant-Microbe Interact..

[42] Wright K.M., Wood N.T., Roberts A.G., Chapman S., Boevink P., MacKenzie K.M., Oparka K.J. (2007). Targeting of TMV Movement Protein to Plasmodesmata Requires the Actin/ER Network; Evidence from FRAP. Traffic.

[43] Ma J., Mudiyanselage S.D.D., Hao J., Wang Y. (2023). Cellular Roadmaps of Viroid Infection. Trends Microbiol..

[44] Zhang Y., Nie Y., Wang L., Wu J. (2024). Viroid Replication, Movement, and the Host Factors Involved. Microorganisms.

[45] Sánchez-Navarro J., Fajardo T., Zicca S., Pallás V., Stavolone L. (2010). Caulimoviridae Tubule-Guided Transport Is Dictated by Movement Protein Properties. J. Virol..

[46] Thomas C., Maule A. (1999). Identification of Inhibitory Mutants of Cauliflower Mosaic Virus Movement Protein Function after Expression in Insect Cells. J. Virol..

[47] Pecher P., Moro G., Canale M.C., Capdevielle S., Singh A., MacLean A., Sugio A., Kuo C.-H., Lopes J.R., Hogenhout S.A. (2019). Phytoplasma SAP11 Effector Destabilization of TCP Transcription Factors Differentially Impact Development and Defence of Arabidopsis versus Maize. PLoS Pathog..

[48] Sugawara K., Honma Y., Komatsu K., Himeno M., Oshima K., Namba S. (2013). The Alteration of Plant Morphology by Small Peptides Released from the Proteolytic Processing of the Bacterial Peptide TENGU. Plant Physiol..

[49] Wei W., Inaba J., Zhao Y., Mowery J.D., Hammond R. (2022). Phytoplasma Infection Blocks Starch Breakdown and Triggers Chloroplast Degradation, Leading to Premature Leaf Senescence, Sucrose Reallocation, and Spatiotemporal Redistribution of Phytohormones. Int. J. Mol. Sci..

[50] Jaouannet M., Magliano M., Arguel M.J., Gourgues M., Evangelisti E., Abad P., Rosso M.-N. (2013). The Root-Knot Nematode Calreticulin Mi-CRT Is a Key Effector in Plant Defense Suppression. Mol. Plant-Microbe Interact..

[51] Jagdale S., Rao U., Giri A.P. (2021). Effectors of Root-Knot Nematodes: An Arsenal for Successful Parasitism. Front. Plant Sci..

[52] Lee C., Chronis D., Kenning C., Peret B., Hewezi T., Davis E.L., Baum T.J., Hussey R., Bennett M., Mitchum M.G. (2011). The Novel Cyst Nematode Effector Protein 19C07 Interacts with the Arabidopsis Auxin Influx Transporter LAX3 to Control Feeding Site Development. Plant Physiol..

[53] Mugford S.T., Barclay E., Drurey C., Findlay K.C., Hogenhout S.A. (2016). An Immuno-Suppressive Aphid Saliva Protein Is Delivered into the Cytosol of Plant Mesophyll Cells during Feeding. Mol. Plant-Microbe Interact..

[54] Elzinga D.A., De Vos M., Jander G. (2014). Suppression of Plant Defenses by a Myzus Persicae (Green Peach Aphid) Salivary Effector Protein. Mol. Plant-Microbe Interact..

[55] Bos J.I., Prince D., Pitino M., Maffei M.E., Win J., Hogenhout S.A. (2010). A Functional Genomics Approach Identifies Candidate Effectors from the Aphid Species *Myzus persicae* (Green Peach Aphid). PLoS Genet..

[56] Morin S., Atkinson P.W., Walling L.L. (2024). Whitefly–Plant Interactions: An Integrated Molecular Perspective. Annu. Rev. Entomol..

[57] van Kleeff P.J., Mastop M., Sun P., Dangol S., van Doore E., Dekker H.L., Kramer G., Lee S., Ryu C.-M., de Vos M. (2024). Discovery of Three Bemisia Tabaci Effectors and Their Effect on Gene Expression in Planta. Mol. Plant-Microbe Interact..

[58] Naalden D., van Kleeff P.J., Dangol S., Mastop M., Corkill R., Hogenhout S.A., Kant M.R., Schuurink R.C. (2021). Spotlight on the Roles of Whitefly Effectors in Insect–Plant Interactions. Front. Plant Sci..

[59] Su Q., Yang F., Hu Y., Peng Z., Huang T., Tong H., Zhang R., Yang Y., Zhou Z., Liang P. (2025). Flavonoids Enhance Tomato Plant Resistance to Whitefly by Interfering with the Expression of a Salivary Effector. Plant Physiol..

[60] Ye W., Yu H., Jian Y., Zeng J., Ji R., Chen H., Lou Y. (2017). A Salivary EF-Hand Calcium-Binding Protein of the Brown Planthopper Nilaparvata Lugens Functions as an Effector for Defense Responses in Rice. Sci. Rep..

[61] Fu J., Shi Y., Wang L., Tian T., Li J., Gong L., Zheng Z., Jing M., Fang J., Ji R. (2022). Planthopper-Secreted Salivary Calmodulin Acts as an Effector for Defense Responses in Rice. Front. Plant Sci..

[62] Hattori M., Nakamura M., Komatsu S., Tsuchihara K., Tamura Y., Hasegawa T. (2012). Molecular Cloning of a Novel Calcium-Binding Protein in the Secreted Saliva of the Green Rice Leafhopper Nephotettix Cincticeps. Insect Biochem. Mol. Biol..

[63] Takagawa M., Yokoyama R. (2025). Current Understanding of the Role of the Cell Wall in Cuscuta Parasitism. Plant Biol..

[64] Shimizu K., Aoki K. (2019). Development of Parasitic Organs of a Stem Holoparasitic Plant in Genus Cuscuta. Front. Plant Sci..

[65] Gaudioso-Pedraza R., Beck M., Frances L., Kirk P., Ripodas C., Niebel A., Oldroyd G.E., Benitez-Alfonso Y., de Carvalho-Niebel F. (2018). Callose-Regulated Symplastic Communication Coordinates Symbiotic Root Nodule Development. Curr. Biol..

[66] Gutjahr C. (2018). Symbiosis: Plasmodesmata Link Root-Nodule Organogenesis with Infection. Curr. Biol..

[67] Plett J.M., Daguerre Y., Wittulsky S., Vayssières A., Deveau A., Melton S.J., Kohler A., Morrell-Falvey J.L., Brun A., Veneault-Fourrey C. (2014). Effector MiSSP7 of the Mutualistic Fungus Laccaria Bicolor Stabilizes the Populus JAZ6 Protein and Represses Jasmonic Acid (JA) Responsive Genes. Proc. Natl. Acad. Sci. USA.

[68] Daguerre Y., Basso V., Hartmann-Wittulski S., Schellenberger R., Meyer L., Bailly J., Kohler A., Plett J.M., Martin F., Veneault-Fourrey C. (2020). The Mutualism Effector MiSSP7 of Laccaria Bicolor Alters the Interactions between the Poplar JAZ6 Protein and Its Associated Proteins. Sci. Rep..

[69] Kloppholz S., Kuhn H., Requena N. (2011). A Secreted Fungal Effector of Glomus Intraradices Promotes Symbiotic Biotrophy. Curr. Biol..

[70] Nizam S., Qiang X., Wawra S., Nostadt R., Getzke F., Schwanke F., Dreyer I., Langen G., Zuccaro A. (2019). Serendipita Indica E5′ NT Modulates Extracellular Nucleotide Levels in the Plant Apoplast and Affects Fungal Colonization. EMBO Rep..

[71] Liu J., Zhang L., Yan D. (2021). Plasmodesmata-Involved Battle against Pathogens and Potential Strategies for Strengthening Hosts. Front. Plant Sci..

[72] Chen X., Li W.-W., Gao J., Wu Z., Du J., Zhang X., Zhu Y.-X. (2024). Arabidopsis PDLP7 Modulated Plasmodesmata Function Is Related to BG10-Dependent Glucosidase Activity Required for Callose Degradation. Sci. Bull..

[73] Lewis J.D., Lazarowitz S.G. (2010). Arabidopsis Synaptotagmin SYTA Regulates Endocytosis and Virus Movement Protein Cell-to-Cell Transport. Proc. Natl. Acad. Sci. USA.

[74] Ishikawa K., Hashimoto M., Yusa A., Koinuma H., Kitazawa Y., Netsu O., Yamaji Y., Namba S. (2017). Dual Targeting of a Virus Movement Protein to ER and Plasma Membrane Subdomains Is Essential for Plasmodesmata Localization. PLoS Pathog..

[75] Wüsthoff K.-P., Steger G. (2022). Conserved Motifs and Domains in Members of Pospiviroidae. Cells.

[76] Zhong X., Leontis N., Qian S., Itaya A., Qi Y., Boris-Lawrie K., Ding B. (2006). Tertiary Structural and Functional Analyses of a Viroid RNA Motif by Isostericity Matrix and Mutagenesis Reveal Its Essential Role in Replication. J. Virol..

[77] Peters W.S., Jensen K.H., Stone H.A., Knoblauch M. (2021). Plasmodesmata and the Problems with Size: Interpreting the Confusion. J. Plant Physiol..

[78] Matilla A.J. (2023). The Interplay between Enucleated Sieve Elements and Companion Cells. Plants.

[79] Wang Y. (2021). Current View and Perspectives in Viroid Replication. Curr. Opin. Virol..

[80] Dissanayaka Mudiyanselage S.D., Ma J., Pechan T., Pechanova O., Liu B., Wang Y. (2022). A Remodeled RNA Polymerase II Complex Catalyzing Viroid RNA-Templated Transcription. PLoS Pathog..

[81] Flores R., Gas M.-E., Molina-Serrano D., Nohales M.-Á., Carbonell A., Gago S., la Peña M.D., Daròs J.-A. (2009). Viroid Replication: Rolling-Circles, Enzymes and Ribozymes. Viruses.

[82] Sugio A., MacLean A.M., Kingdom H.N., Grieve V.M., Manimekalai R., Hogenhout S.A. (2011). Diverse Targets of Phytoplasma Effectors: From Plant Development to Defense against Insects. Annu. Rev. Phytopathol..

[83] Jiang Y., Zhang C.-X., Chen R., He S.Y. (2019). Challenging Battles of Plants with Phloem-Feeding Insects and Prokaryotic Pathogens. Proc. Natl. Acad. Sci. USA.

[84] MacLean A.M., Orlovskis Z., Kowitwanich K., Zdziarska A.M., Angenent G.C., Immink R.G., Hogenhout S.A. (2014). Phytoplasma Effector SAP54 Hijacks Plant Reproduction by Degrading MADS-Box Proteins and Promotes Insect Colonization in a RAD23-Dependent Manner. PLoS Biol..

[85] Orlovskis Z., Singh A., Kliot A., Huang W., Hogenhout S.A. (2025). The Phytoplasma SAP54 Effector Acts as a Molecular Matchmaker for Leafhopper Vectors by Targeting Plant MADS-Box Factor SVP. eLife.

[86] Hoshi A., Oshima K., Kakizawa S., Ishii Y., Ozeki J., Hashimoto M., Komatsu K., Kagiwada S., Yamaji Y., Namba S. (2009). A Unique Virulence Factor for Proliferation and Dwarfism in Plants Identified from a Phytopathogenic Bacterium. Proc. Natl. Acad. Sci. USA.

[87] Gallinger J., Zikeli K., Zimmermann M.R., Görg L.M., Mithöfer A., Reichelt M., Seemüller E., Gross J., Furch A.C. (2021). Specialized 16SrX Phytoplasmas Induce Diverse Morphological and Physiological Changes in Their Respective Fruit Crops. PLoS Pathog..

[88] Bernardini C., Santi S., Mian G., Levy A., Buoso S., Suh J.H., Wang Y., Vincent C., van Bel A.J., Musetti R. (2022). Increased Susceptibility to Chrysanthemum Yellows Phytoplasma Infection in Atcals7ko Plants Is Accompanied by Enhanced Expression of Carbohydrate Transporters. Planta.

[89] Rita M., Alberto L., Rachele P., Karl-Heinz K. (2013). Phytoplasma-Triggered Ca^2+^ Influx Is Involved in Sieve-Tube Blockage. Mol. Plant-Microbe Interact..

[90] Musetti R., Pagliari L., Mian G., De Oliveira Cantao F.R., Bernardini C., Santi S., Van Bel A.J. (2023). The Sieve-Element Endoplasmic Reticulum: A Focal Point of Phytoplasma-Host Plant Interaction?. Front. Microbiol..

[91] Hofmann J., Youssef-Banora M., de Almeida-Engler J., Grundler F.M. (2010). The Role of Callose Deposition along Plasmodesmata in Nematode Feeding Sites. Mol. Plant-Microbe Interact..

[92] Hamamouch N., Li C., Hewezi T., Baum T.J., Mitchum M.G., Hussey R.S., Vodkin L.O., Davis E.L. (2012). The Interaction of the Novel 30C02 Cyst Nematode Effector Protein with a Plant β-1, 3-Endoglucanase May Suppress Host Defence to Promote Parasitism. J. Exp. Bot..

[93] Hewezi T., Howe P.J., Maier T.R., Hussey R.S., Mitchum M.G., Davis E.L., Baum T.J. (2010). Arabidopsis Spermidine Synthase Is Targeted by an Effector Protein of the Cyst Nematode Heterodera Schachtii. Plant Physiol..

[94] Wang J., Replogle A., Hussey R., Baum T., Wang X., Davis E.L., Mitchum M.G. (2011). Identification of Potential Host Plant Mimics of CLAVATA3/ESR (CLE)-like Peptides from the Plant-parasitic Nematode *Heterodera schachtii*. Mol. Plant Pathol..

[95] Wang X., Xue B., Dai J., Qin X., Liu L., Chi Y., Jones J., Li H. (2018). A Novel Meloidogyne Incognita Chorismate Mutase Effector Suppresses Plant Immunity by Manipulating the Salicylic Acid Pathway and Functions Mainly during the Early Stages of Nematode Parasitism. Plant Pathol..

[96] Will T., Furch A.C., Zimmermann M.R. (2013). How Phloem-Feeding Insects Face the Challenge of Phloem-Located Defenses. Front. Plant Sci..

[97] Will T., Kornemann S.R., Furch A.C., Tjallingii W.F., van Bel A.J. (2009). Aphid Watery Saliva Counteracts Sieve-Tube Occlusion: A Universal Phenomenon?. J. Exp. Biol..

[98] Van Bel A.J., Will T. (2016). Functional Evaluation of Proteins in Watery and Gel Saliva of Aphids. Front. Plant Sci..

[99] Pitino M., Hogenhout S.A. (2013). Aphid Protein Effectors Promote Aphid Colonization in a Plant Species-Specific Manner. Mol. Plant-Microbe Interact..

[100] Fischer K., Lachner L.A.-M., Olsen S., Mulisch M., Krause K. (2021). The Enigma of Interspecific Plasmodesmata: Insight from Parasitic Plants. Front. Plant Sci..

[101] Sager R.E., Lee J.-Y. (2018). Plasmodesmata at a Glance. J. Cell Sci..

[102] Kim G., LeBlanc M.L., Wafula E.K., DePamphilis C.W., Westwood J.H. (2014). Genomic-Scale Exchange of mRNA between a Parasitic Plant and Its Hosts. Science.

[103] Hettenhausen C., Li J., Zhuang H., Sun H., Xu Y., Qi J., Zhang J., Lei Y., Qin Y., Sun G. (2017). Stem Parasitic Plant *Cuscuta australis* (Dodder) Transfers Herbivory-Induced Signals among Plants. Proc. Natl. Acad. Sci. USA.

[104] Shahid S., Kim G., Johnson N.R., Wafula E., Wang F., Coruh C., Bernal-Galeano V., Phifer T., Depamphilis C.W., Westwood J.H. (2018). MicroRNAs from the Parasitic Plant *Cuscuta campestris* Target Host Messenger RNAs. Nature.

[105] Hudzik C., Hou Y., Ma W., Axtell M.J. (2020). Exchange of Small Regulatory RNAs between Plants and Their Pests. Plant Physiol..

[106] Alakonya A., Kumar R., Koenig D., Kimura S., Townsley B., Runo S., Garces H.M., Kang J., Yanez A., David-Schwartz R. (2012). Interspecific RNA Interference of SHOOT MERISTEMLESS-like Disrupts *Cuscuta pentagona* Plant Parasitism. Plant Cell.

[107] Spallek T., Melnyk C.W., Wakatake T., Zhang J., Sakamoto Y., Kiba T., Yoshida S., Matsunaga S., Sakakibara H., Shirasu K. (2017). Interspecies Hormonal Control of Host Root Morphology by Parasitic Plants. Proc. Natl. Acad. Sci. USA.

[108] Zeng T., Holmer R., Hontelez J., te Lintel-Hekkert B., Marufu L., de Zeeuw T., Wu F., Schijlen E., Bisseling T., Limpens E. (2018). Host-and Stage-dependent Secretome of the Arbuscular Mycorrhizal Fungus Rhizophagus Irregularis. Plant J..

[109] German L., Yeshvekar R., Benitez-Alfonso Y. (2023). Callose Metabolism and the Regulation of Cell Walls and Plasmodesmata during Plant Mutualistic and Pathogenic Interactions. Plant Cell Environ..

[110] Zeng T., Rodriguez-Moreno L., Mansurkhodzaev A., Wang P., van den Berg W., Gasciolli V., Cottaz S., Fort S., Thomma B.P.H.J., Bono J.-J. (2020). A Lysin Motif Effector Subverts Chitin-Triggered Immunity to Facilitate Arbuscular Mycorrhizal Symbiosis. New Phytol..

[111] Wang P., Jiang H., Boeren S., Dings H., Kulikova O., Bisseling T., Limpens E. (2021). A Nuclear-Targeted Effector of Rhizophagus Irregularis Interferes with Histone 2B Mono-Ubiquitination to Promote Arbuscular Mycorrhization. New Phytol..

[112] Voß S., Betz R., Heidt S., Corradi N., Requena N. (2018). RiCRN1, a Crinkler Effector from the Arbuscular Mycorrhizal Fungus *Rhizophagus irregularis*, Functions in Arbuscule Development. Front. Microbiol..

[113] Sun J., Miwa H., Downie J.A., Oldroyd G.E. (2007). Mastoparan Activates Calcium Spiking Analogous to Nod Factor-Induced Responses in Medicago Truncatula Root Hair Cells. Plant Physiol..

[114] De Storme N., Geelen D. (2014). Callose Homeostasis at Plasmodesmata: Molecular Regulators and Developmental Relevance. Front. Plant Sci..

